# Patients with axial spondyloarthritis reported willingness to use remote care and showed high adherence to electronic patient-reported outcome measures: an 18-month observational study

**DOI:** 10.1007/s00296-024-05673-7

**Published:** 2024-08-21

**Authors:** Emil Eirik Kvernberg Thomassen, Inger Jorid Berg, Eirik Klami Kristianslund, Anne Therese Tveter, Gunnstein Bakland, Laure Gossec, Sarah Hakim, Gary John Macfarlane, Annette de Thurah, Nina Østerås

**Affiliations:** 1 Centre for Treatment of Rheumatic and Musculoskeletal Diseases (REMEDY), Diakonhjemmet Hospital, Oslo, Norway; 2 Faculty of Medicine, University of Oslo (UiO), Oslo, Norway; 3 Department of Rehabilitation Science and Health Technology, Oslo Metropolitan University, Oslo, Norway; 4 Department of Rheumatology, University Hospital of North Norway, Tromsø, Norway; 5 Institute of Clinical Medicine, Faculty of Health Sciences, UiT The Arctic University of Tromsø, Tromsø, Norway; 6 INSERM, Institut Pierre Louis d’Epidémiologie et de Santé Publique, Sorbonne Université, Paris, France; 7 Rheumatology Department Pitié-Salpêtrière Hospital, AP-HP, Paris, France; 8 Aberdeen Centre for Arthritis and Musculoskeletal Health (Epidemiology Group), University of Aberdeen, Aberdeen, UK; 9 Department of Rheumatology, Aarhus University Hospital, Aarhus, Denmark; 10 Department of Clinical Medicine, Aarhus University, Aarhus, Denmark

**Keywords:** Digital health, Axial spondyloarthritis, Rheumatology, Remote monitoring

## Abstract

Remote monitoring using electronic patient reported outcomes (ePROs) in axial spondyloarthritis (axSpA) may improve self-management and reduce the need for consultations. However, knowledge regarding patients’ willingness to use remote care and adherence to reporting ePROs is scarce. The objective of this study was to assess axSpA patients’ willingness to use remote care and adherence to reporting of ePROs. The study was part of a three-armed randomized controlled trial testing digital follow-up strategies (The ReMonit study, NCT: 05031767). AxSpA patients in low disease activity were randomized to usual care, remote monitoring, or patient-initiated care. Demographics, clinical data, and patients’ willingness to use remote care were collected at baseline. EPROs were reported either monthly or quarterly by the remote monitoring- and patient-initiated care group over 18 months, respectively. Adherence to reporting was calculated as number of ePROs completed divided by the total number requested. Mixed model logistic regression was utilized to assess factors associated with adherence to reporting of ePROs. In total 242 patients (median age 43 years, 75% males) were included. The majority (96%) reported high willingness to use remote care. Adherence to reporting ePROs remained high over 18 months by remote monitoring and patient-initiated care groups [median (IQR): 88% (77–100) vs. 83% (66–100)]. No patient characteristics were significantly associated with adherence to reporting of ePROs. The high degree of willingness and adherence to reporting ePROs over time indicates that the majority of axSpA patients with low disease activity are motivated to use remote care.

**Supplementary Information:**

The online version contains supplementary material available at 10.1007/s00296-024-05673-7.

## Introduction

Axial spondyloarthritis (axSpA) is a chronic inflammatory disease of the axial skeleton, which may also affect peripheral joints and have extra-articular manifestations [1]. The disease has significant impact on the patients’ daily life [2], and the management of axSpA is lifelong with regular disease monitoring in order to achieve sustained low disease activity and optimized treatment [3]. During the last decades, advancements in therapeutics have improved treatment outcomes [4]. However, as the disease is characterized by fluctuations in disease activity with episodic flares, continuous monitoring of disease can provide valuable insight both for patients and healthcare providers [5, 6].

The access to timely care may be challenging due to the increasing number of patients and healthcare services facing workforce shortages [7, 8], necessitating alternative ways of delivering adequate care for patients with axSpA [8]. Remote care is an alternative to face-to-face visits that may improve the delivery of care in axSpA patients [9]. While remote care covers a wide range of modalities, the most commonly used modalities include telephone- and video consultations, asynchronous messaging, and regular monitoring of electronic patient-reported outcomes (ePROs) of disease activity [8]. The utilization of remote care and monitoring of ePROs has the potential to enhance patients’ autonomy and allow for more flexible and personalized care [10–12]. Additionally, previous research on remote care has indicated that remote care may be acceptable for rheumatic patients [13].

While the use of remote care and reporting of ePROs could improve access to timely care [12, 14], further studies are needed to investigate axSpA patients’ willingness to use remote care and adherence to reporting of ePROs. Although, previous studies on axSpA patients’ adherence to ePROs have shown promising results over shorter time periods of up to 6 and 12 months [15, 16], studies with longer follow-up are needed. In addition, previous research has investigated adherence to reporting of ePROs on a daily or weekly basis [16, 17]. Frequent reporting of ePROs may be burdensome for patients, and existing evidence is conflicting regarding the optimal frequency of reporting ePROs and how this may affect adherence [17]. Another key component for successful utilization of remote care is to identify potential subgroups of patients with low or high adherence to reporting of ePROs. This may support healthcare providers in identifying patients that may benefit from remote care versus patients who should continue to receive usual face-to-face care.

This study aimed to assess the degree of willingness to use remote care and compare patients’ characteristics across different levels of willingness. Furthermore, this study aimed to examine the adherence to reporting of ePROs over an 18 months follow-up period, and to identify potential subgroups and factors associated with adherence to reporting of ePROs.

## Patients and methods

### Design and setting

Data were collected from patients with axSpA participating in a three-armed non-inferiority randomized controlled trial: remote monitoring of axial spondyloarthritis in specialist healthcare (ReMonit) [18]. The patients were recruited at the outpatient clinic at Diakonhjemmet Hospital between September 2021 and June 2022 and followed over 18 months. All patients provided a written consent to participate in the study.

### Patients

Patients were included according to the inclusion and exclusion criteria in the ReMonit study [18]. In short, patients were included in the trial if they had stable treatment with tumor necrosis factor inhibitors (TNFi) during the last 6 months and had low disease activity defined as Ankylosing Spondylitis Disease Activity score (ASDAS) < 2.1 at inclusion [19, 20].

### Randomization and data collection

At baseline, patients were randomized 1:1:1 to either usual care (face-to-face visit at hospital every 6th month, no reporting of ePRO), remote monitoring (no pre-scheduled visits, monthly reporting of ePROs, monitored by study nurse) or patient-initiated care (no pre-scheduled visits, quarterly reporting of ePROs, not monitored). Patients randomized to remote monitoring and patient-initiated care groups downloaded an app (MyDignio) and were instructed to complete ePROs monthly or quarterly, respectively. Patients in the remote monitoring group were informed that the ePRO data would be routinely monitored. In contrast, the patient-initiated care group was informed that ePROs data would not be monitored, but included in data collection for research purposes. Both groups were instructed on how to contact the study nurse in case of significant disease worsening or adverse events. The app utilized in the study had additional features beyond collecting ePROs, including asynchronous messaging, graphical visualization of ePRO results, automatic reminders to complete ePROs and general information regarding the study. The asynchronous messaging was mostly used for manually reminding patients in case of missed deadlines for reporting ePROs. On average, one manually distributed reminder was sent after each automatic reminder if the patient did not complete the ePRO.

### Measurements

Baseline data on the following variables were collected: age, sex, education level, work status, body mass index (BMI), years since axSpA diagnosis and use of non-steroidal anti-inflammatory (NSAIDs) drugs in the last 6 months prior to inclusion (yes/no). The recommended core outcomes for clinical studies on axSpA [21] were collected digitally at baseline by a patient-reported questionnaire. Disease activity of axSpA was assessed by the ASDAS, Bath Ankylosing Disease Activity Index (BASDAI, Numeric Rating Scale (NRS) 0–10, 0 being best), and Patient Global Assessment of disease activity (PGA NRS 0–10, 0 being best). Self-reported physical function was measured by Bath Ankylosing Spondylitis Functional Index (BASFI NRS 0–10, 0 being best), The Work Productivity and Activity Impairment Index (WPAI NRS 0–10, 0 being best) item 6 was used for assessing disease impact on daily activity. For measuring eHealth literacy, we used four out of seven scales from the eHealth literacy questionnaire (eHLQ), which is a comprehensive questionnaire for assessing eHealth literacy by scoring agreement to a series of statements (0 = strongly disagree, 4 = strongly agree) [22, 23]. The four scales selected were: ‘Using technology to process health information’ (Scale 1), ‘Ability to actively engage with digital services’ (Scale 3), ‘Feel safe and in control’ (Scale 4), and ‘Motivated to engage with digital services’ (Scale 5). All scores were presented as mean and standard deviation (SD) according to eHLQ scoring protocol. In lack of a standardized questionnaire, we developed six questions regarding patients’ self-reported experience in managing different technological equipment such as smartphones, tablets, applications (apps), computers, and digital healthcare services using a 6-point Likert Scale (0 = never tried, 1 = very poor—6 = very good).

Willingness to use remote care in the total sample was assessed at baseline. We developed an ad-hoc statement: “I want to use remote care” where patients rated their agreement on a 4-point Likert scale (1 = strongly disagree, 4 = strongly agree).

For adherence to reporting of ePROs we measured each patient’s completion of ePROs in the app (MyDignio) at each timepoint for the intervention groups (monthly for the remote monitoring versus quarterly for the patient-initiated care group). We calculated the adherence to reporting of ePROs as the patients’ number of completed ePROs divided by the total requested number of ePROs, which is similar to approaches used in previous studies [15, 17, 24]. The ePROs included the PGA and a question regarding if they were experiencing a flare (significant disease worsening) with the response options: “yes”, “no” and “uncertain”. If the patient answered “yes” or “uncertain”, two new questions were trigged regarding the first day (date) and the duration of the flare. Scoring ≥ 3 on the PGA or answering “yes” or “uncertain” on the flare question, prompted the BASDAI-questionnaire.

### Data analysis

Continuous variables are described using either mean and SD or median with inter-quartile range (IQR) according to the distribution of data. Categorical variables are presented as frequency and percentages.

For analysis of willingness to use remote care, all included patients were grouped according to their response on the “willingness to use remote care” item (strongly disagree, disagree, agree, strongly agree). The Kruskal–Wallis equality-of-populations rank test was used to analyze the differences in characteristics between the groups, with Dunn`s post hoc-test if p-values were below alpha-level of 0.05.

The median adherence to reporting of ePROs was calculated for each of the two intervention groups. In addition, the mean percentage (with 95% confidence intervals) of patients in each group answering to the ePROs at each time point was calculated. To test the statistical differences in adherence to ePRO reporting between the remote monitoring and patient-initiated care groups, the Kruskal–Wallis equality-of-populations rank test was applied. Additionally, we categorized the adherence to reporting of ePROs in fixed intervals (80–100, 50–79, 20–40, and 0–19%) and compared median age, percentage of males/females and median BASDAI scores between the different adherence categories.

To investigate baseline characteristics associated with adherence to reporting of ePROs we used a two-level mixed model logistic regression with random intercept. The mixed model was used based on the assumption of clustering in responses of ePROs among patients. We therefore used patients as the random intercept allowing for between-individual baseline variation. For the fixed part of the model, we included the following baseline covariates: age, sex (male/female), eHealth literacy (composite mean score for all four scales), BASDAI, and the number of years since axSpA diagnosis. The covariates included were selected based on prior studies [15, 25, 26], and exploring eHealth literacy’s role in health behavior [27], also as theorized by Norman and Skinner [28] indicating that eHealth literacy is influenced by the disease, educational background and health status. The covariates study group and time (months since baseline) were also included in the fixed part of the model as to adjust for the multiple time-points (18 for the remote monitoring group, and 6 for the patient-initiated care group). Data were analyzed using STATA 17. Significance-level was set to 0.05.

### Ethical considerations

The study was conducted according to the Helsinki declaration and was approved by the Regional Committees for Medical and Health Research Ethics South East Norway (ref: 229187).

### Patient and public involvement

Patient representatives were involved in all stages of this trial, from grant application, development of study materials (including consent procedure, study logistics and questionnaires), interpretation of the results and dissemination of results. Two patient representatives were members of the study project group, of which one (SH) is a co-author of the current article.

## Results

Of the 346 patients that were screened, 242 (70%) were included in the ReMonit study. Patients were randomized to either usual care (n = 82), remote monitoring (n = 79) or patient-initiated care group (n = 81).

**Fig. 1:**
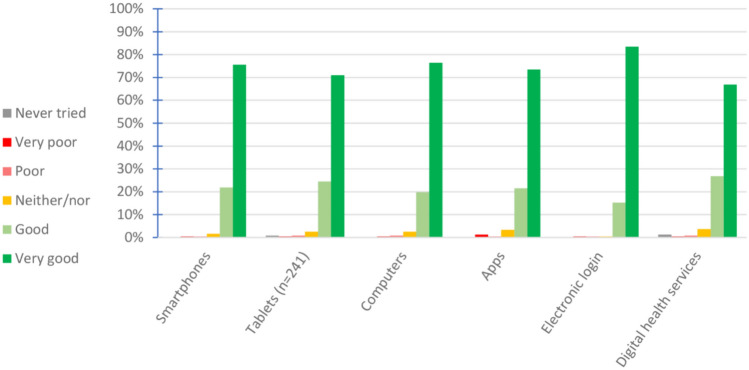
Patients’ experience with different technological equipment (n = 242)

The total sample had a median age of 42 years (IQR: 33–52), and 75% were males. Most patients had university level education and were fully employed at baseline (Table 1). They also had consistently low impact of disease according to the ASDAS, BASDAI, BASFI, and WPAI Item 6 (Table 1). Levels of eHealth literacy were high with scores ranging from 3.3 to 3.6 (Table 1). Patients reported overall high skills in their experience with different technological equipment and digital services, with only a few reporting that they had never used digital health services nor tablets (Fig. 1).

**Table 1:** Baseline demographics and clinical characteristics of patients (n = 242)

Characteristics	Usual care (n = 82)	Remote monitoring (n = 79)	Patient-initiated care (n = 81)	Total (n = 242)
Age, median years (IQR) (min–max)	43 (32–54)	40 (32–48)	43 (38–53)	42 (33–52) (21–76)
Males, n (%)	59 (72%)	61 (77%)	62 (76%)	182 (75%)
Education level^a^, n (%)				
Primary school	3 (4%)	5 (6%)	0	8 (3%)
High school	10 (12%)	6 (8%)	22 (27%)	38 (16%)
University level	68 (84%)	68 (86%)	59 (73%)	195 (81%)
Work status, n (%)				
Full time	68 (83%)	62 (78%)	64 (79%)	194 (80%)
Age retired	5 (6%)	4 (5%)	1 (1%)	10 (4%)
Disability benefit	1 (1%)	2 (3%)	6 (7%)	9 (4%)
Other^b^	8 (10%)	11 (14%)	10 (13%)	29 (12%)
BMI (kg/m^2^), median (IQR)	24.6 (22.3–26.5)	23.9 (22.1–25.9)	25.5 (23.2–28.2)	24.7 (22.6–26.7)
ASDAS-CRP, median (IQR)	0.9 (0.6–1.3)	1.0 (0.7–1.3)	1.0 (0.6–1.4)	0.9 (0.4–1.4)
BASDAI, median (IQR)	1.2 (0.5–2.0)	1.0 (0.5–2.2)	1.0 (0.3–1.7)	1.2 (0.3–2.0)
BASFI, median (IQR)	0.3 (0.0–1.1)	0.3 (0.0–1.3)	0.3 (0.0–1.0)	0.3 (0.0–1.2)
PGA, median (IQR)	1.0 (1.0–2.0)	1.0 (0.0–3.0)	1.0 (0.0–2.0)	1.0 (0.0–2.0)
Pain (NRS), median (IQR)	1.0 (1.0–2)	1.0 (0.0–2.0)	1.0 (0.0–2.0)	1.0 (0.0–2.0)
Morning stiffness, median (IQR)	1.2 (0.0–2.5)	1.5 (0.0–2.5)	1.0 (0.0–2.5)	1.0 (0.0–2.5)
Fatigue, median (IQR)	1.5 (0.0–3.0)	2.0 (0.0–3.0)	1.0 (0.0–3.0)	1.0 (1.0–3.0)
Years since axSpA diagnosis, median (IQR)	11 (5–21)	11 (6–17)	13 (7–20)	12 (6–12)
WPAI item 6, median (IQR)	1.0 (0.0–2.0)	0.0 (0.0–2.0)	1.0 (0.0–2.0)	1.0 (0.0–2.0)
NSAIDs use last 6 months, n (%)	24 (30%)	29 (37%)	27 (33%)	80 (33%)
eHLQ: using technology to process health information^c^, mean (SD)	3.2 (0.5)	3.3 (0.5)	3.1 (0.6)	3.3 (0.6)
eHLQ: ability to actively engage with digital services^d^, mean (SD)	3.5 (0.5)	3.6 (0.4)	3.4 (0.5)	3.6 (0.5)
eHLQ: feel safe and in control^e^, mean (SD)	3.4 (0.5)	3.4 (0.5)	3.3 (0.4)	3.4 (0.5)
eHLQ: Motivated to engage with digital services^f^, mean (SD)	3.3 (0.5)	3.3 (0.5)	3.1 (0.6)	3.3 (0.5)

*IQR* inter quartile range, *SD* standard deviation

^a^n = 241 due to 1 missing

^b^Other: receiving social benefits, on sick leave, student/housekeeping, part time work and unemployed, ASDAS: Ankylosing spondylitis Disease Activity Score, BASDAI: Bath Ankylosing Spondylitis Disease Activity Index, BASFI: bath ankylosing spondylitis functional index (0–10, 10 being worst), Patient global assessment of disease activity (0–10, 10 being worst), WPAI Item 6: Work Productivity and Activity Impairment: Ability to perform daily activities (NRS 0–10, 10 being worst), NSAIDs use last 6 months: non-steroidal anti-inflammatory drugs, eHLQ (0–4, 4 being best): eHealth literacy Questionnaire

^c^Scale 1: using technology to process health information

^d^Scale 3: ability to actively engage with digital services

^e^Scale 4: feel safe and in control

^f^Scale 5: motivated to engage with digital services

**Fig. 2:**
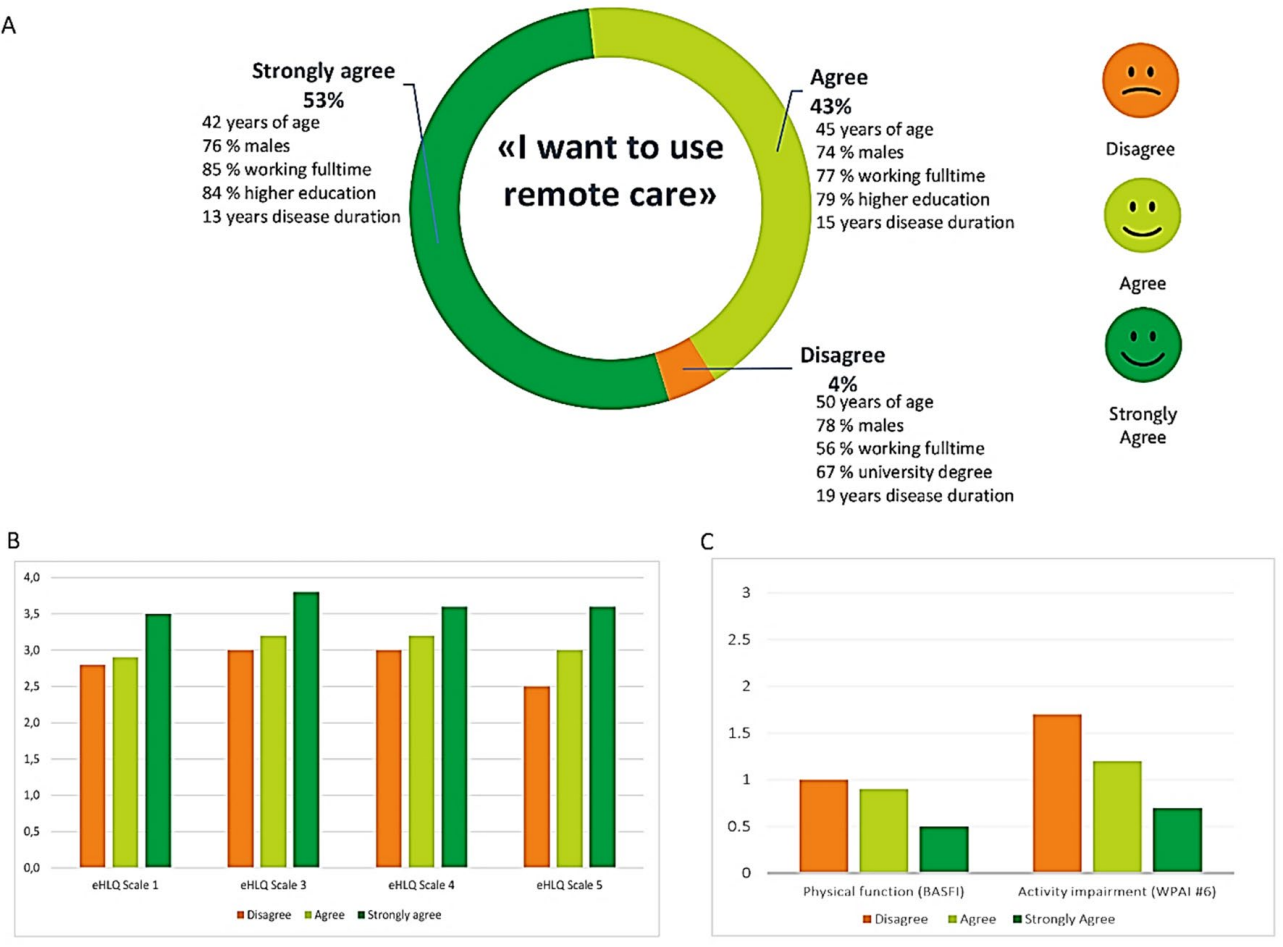
Characteristics of patients with different levels of willingness to use remote care (n = 242)

The single-item question regarding willingness to use remote care showed that 233 of 242 (96%) patients reported that they agreed to or strongly agreed to use remote care (Fig. 2). The patients with the highest level of willingness (“strongly agree”) were significantly younger of age, had a higher level of education, more likely to be full-time employed, and had better physical function and higher levels of eHealth literacy compared to patients who either agreed or disagreed to using remote care (Fig. 2).

**Fig. 3:**
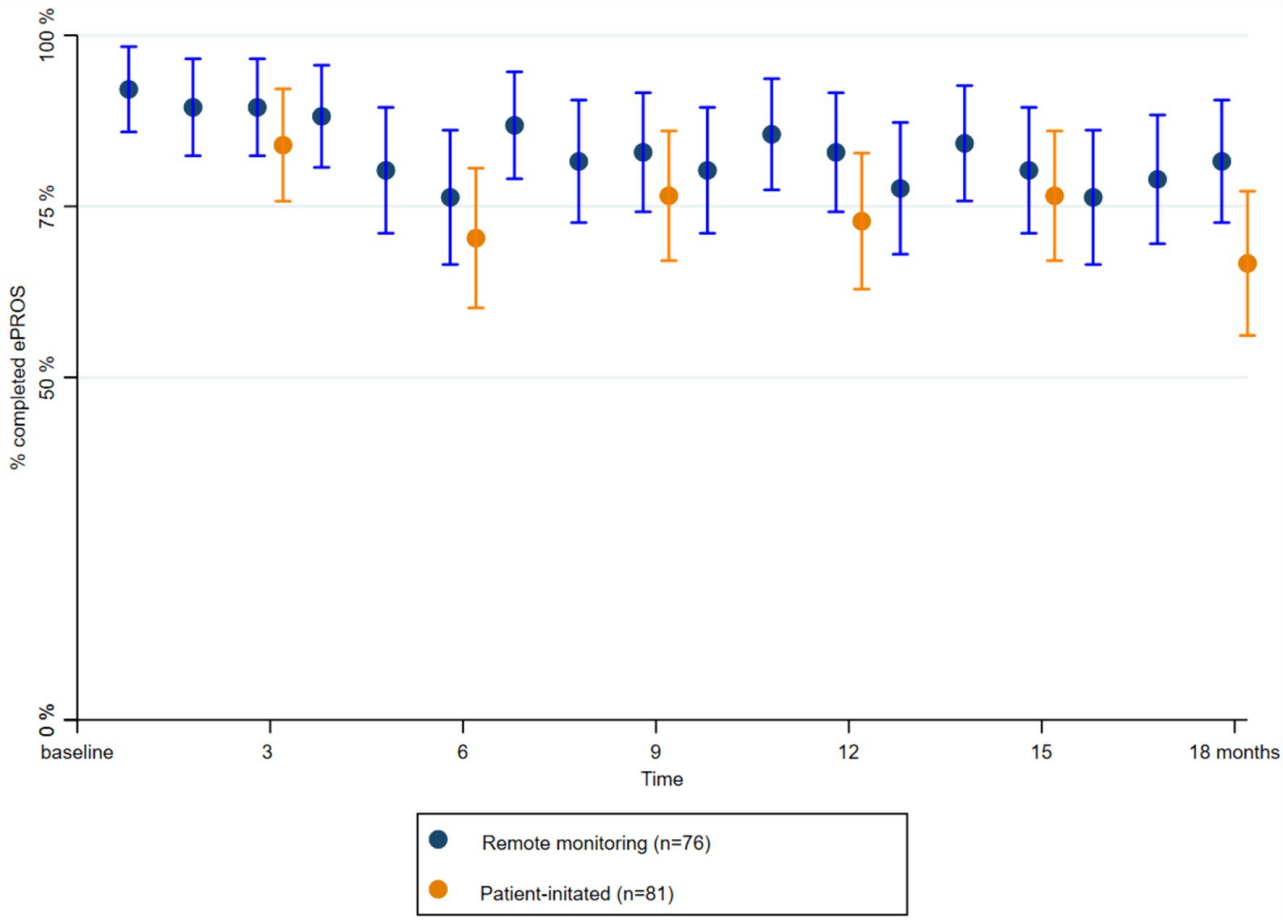
Estimated mean percentage of completed ePROs by time-points with 95% confidence intervals in the remote monitoring group (blue) and the patient-initiated care group (orange) (n = 76 vs. n = 81)

The adherence to self-reporting of ePROs was high in both the remote monitoring and patient-initiated care group. While the adherence decreased somewhat at the 6-month time-point, it was followed by stable reporting in the subsequent time-points (Fig. 3). There was no statistically significant between-group difference in median adherence to reporting of ePROs (remote monitoring, median: 88%, IQR: 77–100%; patient-initiated care, median: 83, IQR: 66–100%; p = 0.428). Automatic reminders were sent out to 133/160 (84%) patients counting a total of 836 automatically generated reminders.

**Fig. 4:**
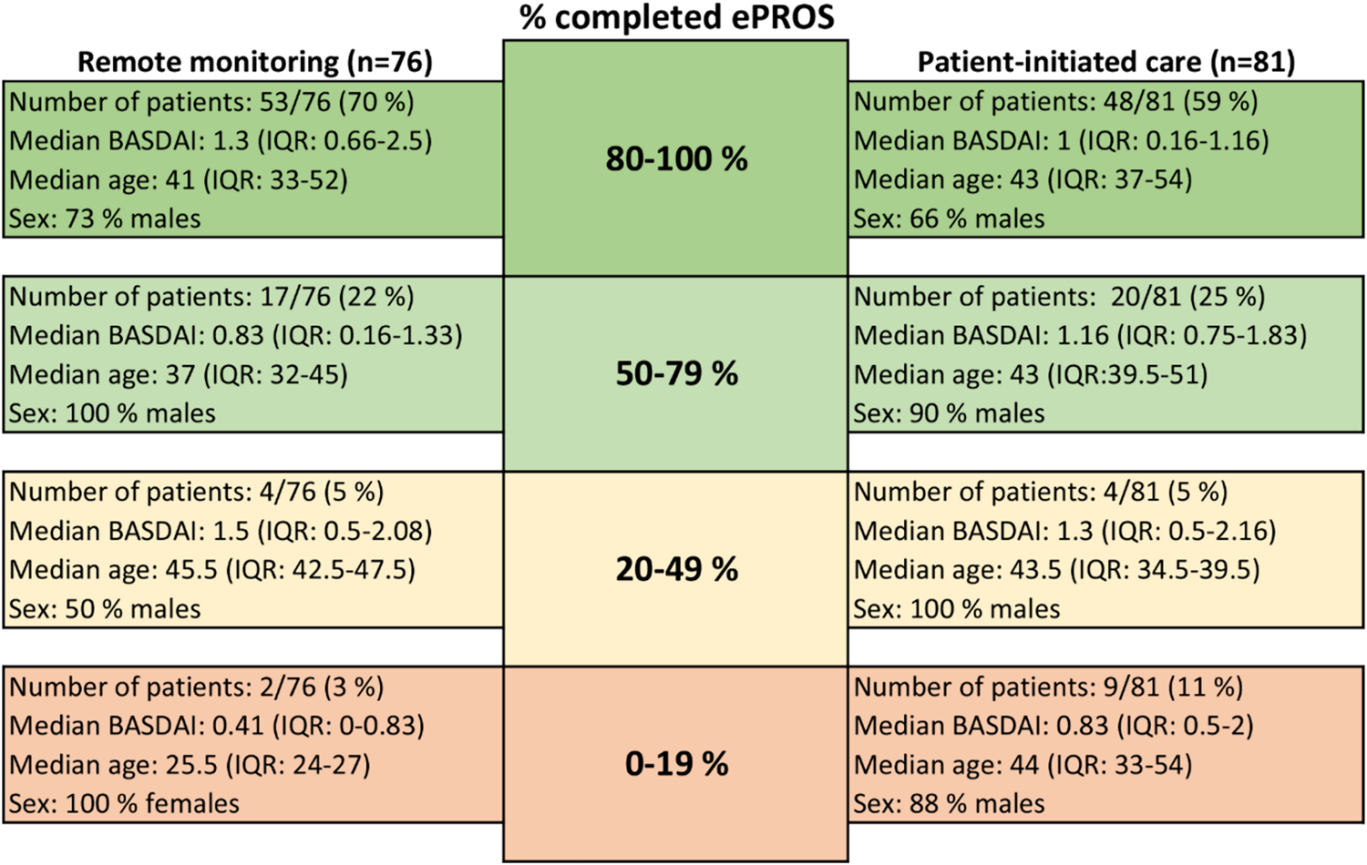
Categorization of different levels of adherence to reporting of electronic patient-reported outcomes (ePROs) with characterizations of self-reported disease activity (BASDAI), age, and sex (n = 157)

The majority of patients in both intervention groups demonstrated between 80–100% adherence to reporting of ePROs (Fig. 4). In the remote monitoring group, the patients completing between 50–79% of the ePROs were slightly younger and had a lower BASDAI compared to the other adherence categories within the same group. No major differences were seen across categories in the patient-initiated care group. Notably, five patients in the patient-initiated care group did not complete any of the ePROs during the 18-month follow-up period (Supplementary file).

The two-level mixed model was inconclusive in showing associations between baseline variables and adherence to reporting of ePROs over the 18-month period (Table 2). In the crude model, higher age and higher disease activity showed a slightly higher odds of patients answering to the ePROs, but no significant associations were retained in the adjusted model.

**Table 2:** Factors associated with adherence to reporting of electronic patient-reported outcomes (ePROs) (n = 157)

Dependent variable: completed ePRO (yes/no)	Crude model			Adjusted model		
Variable	OR	[95% CI]	P-value	OR	[95% CI]	P-value
Age	1.08	1.00, 1.02	0.001	1.04	0.99, 1.08	0.090
Sex (female)	0.91	0.69, 1.19	0.497	1.31	0.54, 3.18	0.542
eHealth literacy	1.10	0.85, 1.44	0.440	2.03	0.81, 5.11	0.130
Disease activity	1.17	1.05, 1.31	0.004	1.06	0.72, 1.51	0.715
Years with axSpA	1.01	0.99, 1.02	0.091	0.98	0.94, 1.03	0.491

Mixed effects logistic regression with random effects adjusted for group and time (months)

OR = odds ratio, eHealth literacy: mean scores of scales 1, 3, 4 and 5 from the eHealth literacy questionnaire, Disease activity: measured by Bath ankylosing spondylitis Disease Activity Index (BASDAI)

## Discussion

This study showed that axSpA patients with low disease activity reported a high willingness to use remote care. Patients who reported the highest degree of willingness were more likely to be younger, have higher education, be full-time employed, have better physical function and higher eHealth literacy as compared to those reporting a lower willingness. The adherence to reporting of ePROs over 18 months was high regardless of monthly or quarterly reporting of ePROs. In the adjusted multivariate analysis, none of the patient characteristics or clinical characteristics at baseline were associated with adherence to reporting of ePROs.

Although the high willingness to use remote care is based on a single item, the high motivation for engaging with digital services on the eHLQ-scale points in the same direction. High willingness is also reported in previous studies in patients with inflammatory joint diseases showing readiness to use remote care given proper access to technology [29, 30].

The high adherence to reporting of ePROs in this study is in accordance with a previous report showing a pooled average of 80% adherence in studies on adherence to reporting ePROs among patients with inflammatory arthritis [17]. Our study showed that regardless of different time intervals for reporting ePROs, the adherence sustained over the 18 months follow-up period. This is in contrast to most studies showing a decline in adherence over time [17]. Given our study’s long follow-up period, we anticipated that a decline would occur, but this was not seen. Different types of reminders for the patients in our study, both push-notifications (within the smartphone operative system), regular text-messages, and reminders sent via the chat-function in the app may have counteracted a decline. Although the effect of reminders on adherence is unclear [25, 31], it may have had a positive effect on patients’ adherence to reporting of ePRO in our study. Furthermore, as this study was conducted as a randomized controlled trial with follow-up at 6, 12, and 18 months, the completion of questionnaires at these follow-up time-points may have served as additional reminders and contributed to an increased adherence to reporting of ePROs. Moreover, the high motivation to use remote care, both demonstrated by the high willingness to use remote care and the high motivation to engage with digital services, may explain the consistently high adherence.

The current study provides evidence for high adherence to reporting of ePROs with longer intervals, i.e., quarterly reporting. It has previously been suggested that in patients with stable treatment and low disease activity, shorter intervals of ePROs will most likely result in lower adherence, as this may be perceived as too burdensome in proportion to the burden of their disease [25, 32, 33]. However, fluctuations of pain are also frequent among patients with low to moderate disease activity [34], which might be an argument for shorter intervals in ePROs monitoring.

In our study, we did not find any statistically significant associations between adherence to reporting of ePROs and baseline characteristics. The lack of associations is in line with the results from a systematic review on adherence to reporting of ePROs in patients with other chronic conditions [35]. In contrast, another study found that women were more likely to discontinue reporting of ePROs [15]. Comparable to our study, this study also failed to find any associations between adherence and age or disease duration [15]. Furthermore, Jones et al. [36] showed that covariates such as time of reporting of ePROs, age, disease-related symptoms, smartphone operating system, use of an activity tracker, and mean pain rating explained 27% of the variance of daily adherence to reporting of ePROs. The inconclusive and uncertain results concerning factors influencing adherence may indicate that the construct of adherence to reporting of ePROs (or PROs in general) is a complex behaviour which is not fully understood [24].

Our study showed that a large number of patients needed the automatic reminders throughout the study period in order to remember to complete the ePROs. These reminders prompted an alert which had to be managed by the project group. The increased workload introduced by managing alerts, could potentially act as a barrier among healthcare professionals for future implementation of remote care.

Technical problems could be another possible barrier for future utilization of remote care [37]. However, after the 18 months follow-up we only excluded one patient from adherence analysis due to malfunction of the app. Thus, there was a low amount of technical errors in the monitoring of ePROs in this study.

Lastly, the potentially negative impact of using ePROs in remote monitoring should be reflected upon as the frequent monitoring could act as regular reminders of their rheumatic disease. As found in a similar study, the experiences with using ePROs provoked a sense of negative emotions as the completion of ePROs could highlight the patients’ functional limitations [36].

The strengths of our study include a longer follow-up time as compared to previous studies, but future studies should have longer follow-up to assess adherence beyond 18 months. Another strength is the testing of both monthly and quarterly reporting, which provides knowledge of the adherence to different intervals of ePRO reporting. A possible limitation in our study is that both Android and iOS have automatic settings in which infrequent-used apps are put into “sleep mode”. This may have led to fewer push notifications and reminders from the app. As we also sent reminders as text-messages, this may have compensated for push-notifications not reaching patients. Another potential limitation to the measurement of adherence to reporting of ePROs is that the patient-initiated care group were told that the data would not be monitored, which may have reduced their motivation for answering. However, the results do not indicate a lack of motivation for reporting ePROs among this group compared to the remote monitoring group.

Studies on remote care are in general prone to selection bias since most of the studies solely include patients with access to smartphones or computers alongside sufficient skills using them [17]. As we included patients from a randomized controlled study willing to test remote care, this might have led to an overrepresentation of patients with an interest in receiving remote care. Hence, we cannot disregard a selection bias in our study as the patients reported both high levels of eHealth literacy, good experience in using different electronic devices, high education level, and low disease activity. This might also have led to skewness in adherence to reporting of ePROs in the current study as patients unwilling to participate may have had lower adherence. On the other hand, as only 30% of the screened patients did not fulfil the inclusion criteria or were unwilling to participate in the study, this may reduce the likelihood of a selection bias.

The large proportion of the sample in the current study had university-level education, which may weaken the external validity of the adherence results as higher socio-economic status has been stated as a potential predictor of adherence to reporting ePROs among patients with rheumatoid arthritis [38]. The patients in our study also had low disease activity and minimally disease-impact in their daily life, which means that caution should be applied when generalizing the results to other patients. Additionally, the relatively long average disease duration in this study sample could suggest that results may not fully apply to people who are newly diagnosed with axSpA. However, as we included 70% of the patients that were screened, we believe that the included sample may be a representative sample of axSpA patients with low disease activity.

In conclusion, this study showed a high willingness to use remote care among patients with axSpA with low disease activity and demonstrated high adherence to reporting of ePROs over a period of 18 months.

## Supplementary Information

Below is the link to the electronic supplementary material.

Supplementary file1 (DOCX 1021 KB)

## Data Availability

The data utilized in analysis of this study are not openly available due to reasons of sensitivity and regulations.
