# Supplementary material for: Patients with axial spondyloarthritis reported willingness to use remote care and showed high adherence to electronic patient-reported outcome measures: an 18-month observational study
Source: Rheumatol Int. 2024 Aug 21;44(10):2089–98. doi: 10.1007/s00296-024-05673-7 (PMC11393250; doi:10.1007/s00296-024-05673-7)

**Supplementary materials Figure 1**. Heatmap displaying patterns of response to ePROs during 18 months among patients in the remote monitoring group (n=79)


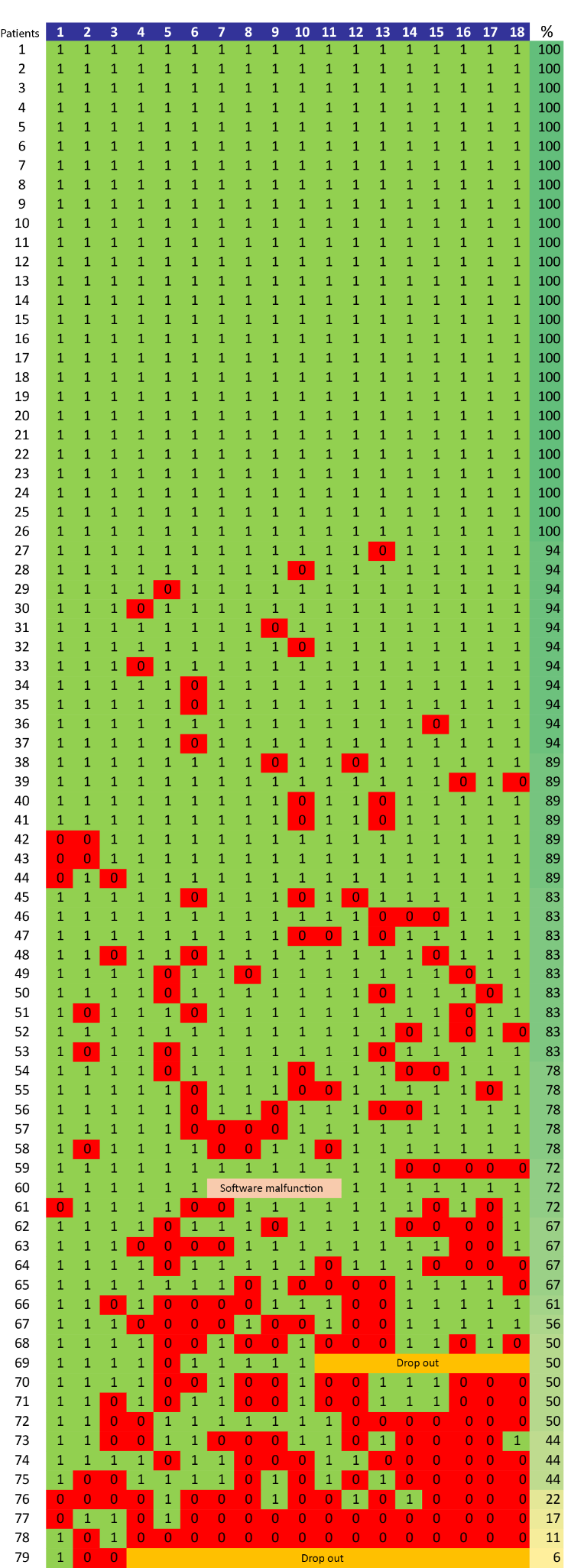


**Figure 2**. Heatmap displaying patterns of response to ePROs during 18 months among patients in the patient-initiated group (n=81)


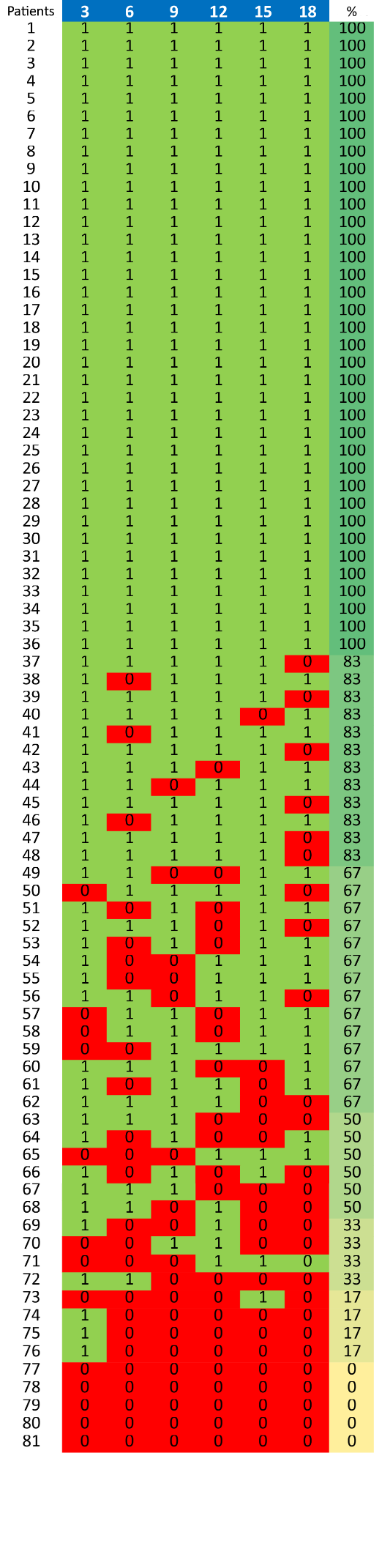

Supplement: Supplementary file 1 — Supplementary file1 (DOCX 1021 KB) [file 296_2024_5673_MOESM1_ESM.docx]
